# Severe Microbial Keratitis Secondary to Prostaglandin-Associated Periorbitopathy

**DOI:** 10.1155/crop/5635118

**Published:** 2025-10-08

**Authors:** Matthew H. McCartney, Fady K. Sammouh, Jessica Y. Tong, Mark Chehade, Dinesh Selva

**Affiliations:** ^1^Department of Ophthalmology & Visual Sciences, University of Adelaide, Adelaide, Australia; ^2^Department of Ophthalmology, Royal Adelaide Hospital, Adelaide, Australia

**Keywords:** bimatoprost, eyelid diseases, latanoprost, microbial keratitis, prostaglandin-associated periorbitopathy, travoprost

## Abstract

Prostaglandin-associated periorbitopathy (PAP) is a known complication of prostaglandin therapy most commonly associated with topical bimatoprost. PAP is characterized by a progressive constellation of eyelid, periorbital, and conjunctival signs that contribute to cosmetic and functional challenges for patients and clinicians. This constellation of findings can produce an asymmetric appearance, symptomatic discomfort, and ocular surface changes to patients. In our case, these changes resulted in previously unreported sight-threatening sequelae from bacterial keratitis. The authors report a case of PAP-induced lagophthalmos and secondary microbial keratitis that necessitated a corneal graft. Reversal of these findings occurred on prostaglandin cessation with dramatic reversal of findings within 8 weeks.

**Summary**

• The authors present a case of reversible, prostaglandin-associated periorbitopathy (PAP)–induced lagophthalmos with secondary microbial keratitis necessitating corneal grafting.

## 1. Introduction

PAP is a known complication of topical prostaglandin therapy most commonly associated with bimatoprost [1]. PAP, coined by Berke [1, 2], is characterized by upper lid ptosis, deepening of upper lid sulcus, dermatochalasis, periorbital fat atrophy, enophthalmos, inferior scleral show, prominence of lid vessels, and tight lids. Prospective cross-sectional surveys have shown PGA users demonstrated a 230-fold increased risk of involution of dermatochalasis and a 249-fold increased risk of lower lid fat atrophy, with effects detectable within 4 weeks of treatment induction [3].

Grading systems have been proposed to highlight the progressive nature of PAP that can occur over time, including the recently published Shimane University prostaglandin-associated periorbitopathy (SU-PAP) system [4]. The grading of PAP ranges from 0 (no evidence) to Grade 1 (lash growth or skin pigmentation), Grade 2 (cosmetic changes and deep upper lid sulci, enophthalmos, fat atrophy, or dermatochalasis involution), and Grade 3 (difficulty performing applanation tonometry secondary to PAP findings, hardening of lids, ptosis) [4].

The authors present a case study of a patient with severe progressive PAP with lagophthalmos and eventual development of severe microbial keratitis necessitating corneal grafting. Following cessation of the PGA, the patient's lagophthalmos on forced closure resolved after 8 weeks, and no further surgical management was necessary. This significant improvement achieved highlights the benefits of PGA cessation and should be considered as part of first-line management, given surgical management of PAP-related sequelae can be difficult due to its progressive nature and therefore unpredictable treatment response. This study was conducted in accordance with the Declaration of Helsinki, and informed, written consent for publication was provided by the patient.

## 2. Case Presentation

An 80-year-old male developed a 1-week history of epiphora and redness in the left eye but did not seek medical care. At the time of a planned excisional biopsy for a suspicious limbal lesion, a left microbial keratitis was identified, and the patient was admitted for further assessment and treatment.

The patient's ophthalmic history included bilateral pseudophakia and primary open angle glaucoma being treated with combination travoprost/timolol nocte to both eyes for 10 years. There was no relevant past medical history.

**Figure 1:**
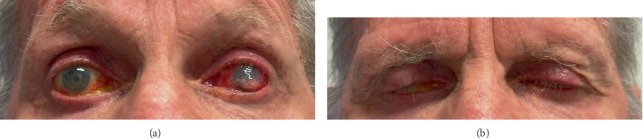
Clinical appearance at presentation: Facial photographs on maximal lid opening (a) and maximal lid closure (b). Evident lagophthalmos, bilateral deepened upper lid sulci, involution of age-related dermatochalasis, periorbital fat atrophy, and prominent, injected vessels of the upper and lower lids and conjunctiva with associated anterior blepharitis. Evident left eye inferior microbial keratitis.

Periorbital examination demonstrated bilateral changes consistent with Grade 3 PAP (using the SU-PAP grading system) [4] including bilateral deepened upper lid sulci, periorbital fat atrophy, bilateral mild lid retraction, tight eyelids, and prominent, injected vessels of the upper and lower lids and conjunctiva (Figure 1). There was also evidence of bilateral lagophthalmos on forced closure of 4 mm in both eyes, with a reduced Bell's reflex inducing inferior corneal exposure.

The right cornea was clear with demonstrable evidence of inferior corneal neovascularization and scarring consistent with chronic exposure keratopathy. The left eye demonstrated multiple stromal infiltrates with an overlying 6 × 10 mm epithelial defect and approximately 30% loss of stromal bed with a mixed hypopyon/hyphema and no posterior segment view. A corneal scrape was undertaken (culturing *Moraxella nonliquefaciens* and *Staphylococcus epidermidis*), and the patient was commenced on gentamycin 1.4% and cefazolin 5% drops hourly as well as oral ciprofloxacin 500 mg BD, vitamin C, and doxycycline 100 mg mane.

The patient was evaluated by the oculoplastics service on Day 7 of admission in relation to his lagophthalmos and possible requirement for a tarsorrhaphy. Given the lack of corneal surface breakdown or epitheliopathy progression, the decision was made to change the patient from combination travoprost/timolol to dorzolamide/timolol and continue lubricants.

The dense corneal infiltrate and associated thinning, after corneal review, necessitated a tectonic penetrating keratoplasty undertaken on Day 10 postpresentation.

**Figure 2:**
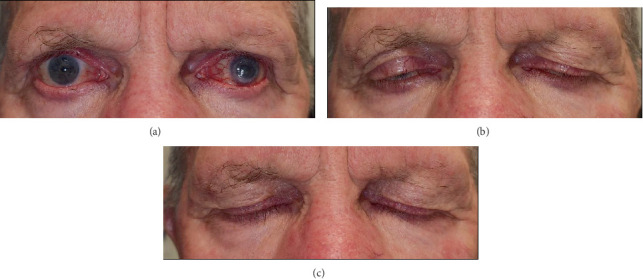
Clinical appearance at 8 weeks post travoprost cessation. Facial photographs on maximal lid opening (a), voluntary blink (b), and maximal lid closure (c). Notably, there is no lagophthalmos on forced closure, reversal of dermatochalasis involution, and some reversal of periorbital fat atrophy, most apparent in the lower lid fat pads.

At 8 weeks post presentation, the patient demonstrated no lagophthalmos on forced blink, with reduction in the severity of PAP to Grade 2 (Figure 2). The conjunctival and eyelid redness were significantly reduced, and the relative laxity of both upper and lower lids had increased with some recovery of periorbital fat atrophy. The IOP remained below target pressure in the context of their treatment regimen change.

## 3. Discussion

The authors present a case demonstrating the severe potential effects of progressive PAP to highlight rare sight-threatening sequelae and the remarkable effects of PGA cessation. In this case, progressively worsening ocular surface (mostly from exposure) set the scene for refractory microbial keratitis, eventually requiring a tectonic corneal graft. The incidence of PAP in PGA use is high, with rates reported to be 93.3% of bimatoprost users, 41.4% of latanoprost users, and 70% of travoprost users [5].

Histological examination of orbital fat in PGA users has demonstrated increased adipocyte density with clumped nuclei suggestive of atrophy [6]. Activation of the FP-prostanoid receptor by PGAs downregulates expression of peroxisome proliferator-activated receptor gamma (PPAR-gamma), thereby inhibiting accumulation of lipid droplets [7]. This inhibitory effect is agent dependent; bimatoprost was shown to have the greatest effect, followed by travoprost, with latanoprost downregulating the least [7]. PGAs also alter matrix metalloproteinase (MMP) expression, causing potential levator palpebrae superioris dehiscence and/or Muller's muscle fibrosis, resulting in either ptosis or lid retraction [8].

The extent of clinical effects of PAP on lid function is not completely understood, and further associations continue to be identified. Lagophthalmos from severe PAP has been previously reported over case series and cohort studies; Rabinowitz et al. reviewed a cohort of 33 patients on monocular PGA therapy for 12 months and found a statistically significant increase in lagophthalmos, upper lid retraction, and eyelid redness [8]. In addition, lagophthalmos and lid redness were significantly increased in patients on bimatoprost when compared to latanoprost and travoprost users [8]. The study also used a novel grading system for PAP, utilizing the degree of fat atrophy and superior sulcus deformity by measuring the relationship of the superior sulcus to the orbital rim. Grade II disease, defined as fat atrophy, with upper lid dermatochalasis involution and no posterior migration of the superior sulcus, was associated with the greatest amount of lagopthalmos [8]. Rabinowitz et al. hypothesized that lagophthalmos occurs due to a combination of inflammation, levator fibrosis/tightening, and sympathetic innervation alteration causing Muller's muscle overaction [8]. In addition, PGAs have been demonstrated to worsen meibomian gland functional parameters, reduce tear-film break up time, and worsen ocular surface staining by Oxford grading, all of which increase the risk of keratitis formation [4].

Preservatives in preserved drops can cause additional ocular surface toxicity as well. In our case, the patient was in fact using preserved eye drops, which could have caused further toxic keratoconjunctivitis. However, the location of exposure keratitis and correlation to orbital findings show that PAP was the main culprit.

The first-line management of PAP is to discontinue the inciting agent where possible. Reversal of some PAP changes is often seen within 4–6 weeks, with the most common changes being the return of previously absent dermatochalasis and shallowing of the upper lid sulci [9]. However, the resolution of changes may be incomplete, and reversal can continue for as long as 24 months and may not recover in severe cases [9]. Patients may also be changed to a PGA with lesser PAP-inducing effects, such as latanoprost, if discontinuation is not an option. Botulinum toxin to induce a therapeutic ptosis as well as suture tarsorrhaphy can be used as temporizing measures [2, 5, 6].

Newer PGA agents with more selective binding sites demonstrate promising results in reducing the incidence of PAP. Omidenepag isopropyl is a PGA that selectively binds the G protein-coupled receptor prostaglandin-EP2 and is currently undergoing Phase III trials. It reduced PAP signs in almost half of the patients previously being treated with conventional PGA [10].

This is the first report to document and illustrate how severe and serious PAP could be, requiring a penetrating keratoplasty and multidisciplinary approach. PAP is a phenomenon that may be reversed with alteration of a patient's drop regimen. If left undetected, progressive change can have sight-threatening manifestations, as presented. Early recognition and action are paramount. Newer agents present promising alternatives with potentially less likelihood of PAP incidence.

## Data Availability

The data that support the findings of this study are available from the corresponding author upon reasonable request.
